# The New Protective Power against Infectious Diseases

**Published:** 1907-03

**Authors:** 


### The New Protective Power Against Infectious Diseases.

---

Now we have a new theory added to the many regarding the antitoxic treatment of infectious diseases. Heretofore antitoxin in itself was supposed to explain everything so far as the counteraction of a special poison was concerned. By and by it was discovered that immunity was really due to a very complicated process in the blood whereby numerous so-called anti-bodies were produced which acted conjointly in overcoming the enemy.

As it was conceded that the poison, or toxin, given off by the bacteria did the principal mischief, the aim was to neutralize these products by some opposing agent that would make the individual ultimately immune.

The theory of the treatment of diphtheria is based on this assumption. By inoculating a horse with the diphtheria virus in doses of progressive strength the resistance of the animal to the poison finally became such that he ceases to react against the most virulent dose. Then his filtered blood containing the developed antitoxin is transferred to the diphtheria victim, who was thus transformed into a likewise immunized condition.

But whatever way this was actually accomplished the practical fact remained that the diphtheritic patients were ultimately cured by the treatment. The discovery of the serum for the treatment of tetanus was also based on the same principle.

The reason why some individuals escaped contagion or affection was explained by the assumption that the suitable antitoxin of the blood were already there in sufficient quantities to ward off the attack. Hence such was naturally immune.

It was also contended that these anti-bodies which combated the poisons in the blood not only neutralized the poisons given off by the various intruding bacteria, but destroyed the bacteria themselves.

Besides this it was claimed by Metchnikoff, the present illustrious chief of the Pasteur Institute of Paris, that the bacteria were demolished by the special action of the white, or lymph, corpuscles of the blood — that is, actually devoured by them — and hence the significant term of phagocytes was applied to them. He demonstrated this action by various convincing experiments, and his theory, so far as it goes, is quite universally accepted by the various scientific observers here and abroad.

Now it comes about to explain why these phagocytes do not attack the enemy by some given rule, for in some cases it is admitted that they are either insufficient in number or do not act to any desirable purpose.

Of course it was necessary to show the why and wherefore. The theorists then came to the front again and much argument was expended, with the result of making confusion worse confounded. Professor Ehrlich of Germany, launched his claim theory of "infection and immunity," which, ingenious and logical as it appears, is far from accounting for all the phenomena and tended not a little to add to their complexity.

But all this time the scientists have been making due progress. At last they have discovered an entirely new vital property in the blood, which they claim is of the utmost importance in reconciling all previous difficulties in explaining the protective power of immunity. This new substance is opsonin. As its name indicates it is a caterer. In other words, it is supposed by its action on the bacteria to give them sufficient flavor or other preparation to tempt the phagocytes, or devouring white blood corpuscles, to do their full duty.

If by examining the blood it is discovered that there is enough opsonin present to insure the destruction of a maximum amount of bacteria, the individual is considered to have good opsonic power. If not, the aim is to develop such power by vaccinating the infected individual with the dead bacteria of the special disease and thus increase his so-called opsonic protection against further inroads.

So far limited experimentation is very encouraging. Allowing the theory to be correct, the newly discovered opsonic power of the individual may lie at the bottom of all prevention and cure of infectious diseases.—*New York Herald.*

